# THE HERO CARE SURVEY: MEASURING THE UNMET NEEDS OF AMERICAN MILITARY VETERANS AND THEIR CAREGIVERS

**DOI:** 10.1093/geroni/igae098.1091

**Published:** 2024-12-31

**Authors:** Stuti Dang, Ranak Trivedi, Luci Leykum

**Affiliations:** Chair; Co-Chair; Discussant

## Abstract

Success in delaying long term institutional care (LTIC) depends on identifying unmet needs of Veteran-caregiver dyads and creating strategies to adequately support those needs. This symposium aims to enhance the understanding of the needs of Veterans and caregivers at high LTIC risk, to inform better planning of services and policy for Veterans and their caregivers. Our interdisciplinary group of researchers will use data from our prospective HERO CARE survey, fielded to 20,000 community-dwelling high-need, high-risk Veterans and their caregivers, to report on the epidemiology of their needs over time. We will review the survey’s socio-ecological framework; summarize survey responses from Veterans and caregivers (Dang, Chair), followed by an overview of the VA-funded Elizabeth Dole National Center for Veteran and Caregiver Research (EDCoE), under whose umbrella this survey was conducted. (Co-chair-Trivedi). We will describe unmet needs stratified by LTIC risk and compare racial/ethnic differences in the prevalence of unmet needs. (Garcia) The next speaker will describe the process of analysis of HERO CARE caregivers’ self-perceived preparedness for caregiving, awareness of resources and desire to institutionalize. (Desir) We will then describe key characteristics and preliminary findings for Veterans who report having a caregiver younger than age 25, an often-overlooked demographic of caregivers. (Kalvesmaki) Our last speaker will report on unmet needs and physical and emotional health of caregivers as they transition to survivors after the death of their care-recipient. (Bouldin) Finally, the discussant will synthesize the presentations and potential policy implications of the findings. (Leykum)

